# Remembering that big things sound big: Sound symbolism and associative memory

**DOI:** 10.1186/s41235-016-0047-y

**Published:** 2017-02-20

**Authors:** Melissa A. Preziosi, Jennifer H. Coane

**Affiliations:** Colby College, 5550 Mayflower Hill, Waterville, ME 04901-8855 USA

**Keywords:** Congruency Effect, Forced Choice Task, Size Judgment, Size Descriptor, Front Vowel

## Abstract

According to sound symbolism theory, individual sounds or clusters of sounds can convey meaning. To examine the role of sound symbolic effects on processing and memory for nonwords, we developed a novel set of 100 nonwords to convey largeness (nonwords containing plosive consonants and back vowels) and smallness (nonwords containing fricative consonants and front vowels). In Experiments 1A and 1B, participants rated the size of the 100 nonwords and provided definitions to them as if they were products. Nonwords composed of fricative/front vowels were rated as smaller than those composed of plosive/back vowels. In Experiment 2, participants studied sound symbolic congruent and incongruent nonword and participant-generated definition pairings. Definitions paired with nonwords that matched the size and participant-generated meanings were recalled better than those that did not match. When the participant-generated definitions were re-paired with other nonwords, this mnemonic advantage was reduced, although still reliable. In a final free association study, the possibility that plosive/back vowel and fricative/front vowel nonwords elicit sound symbolic size effects due to mediation from word neighbors was ruled out. Together, these results suggest that definitions that are sound symbolically congruent with a nonword are more memorable than incongruent definition-nonword pairings. This work has implications for the creation of brand names and how to create brand names that not only convey desired product characteristics, but also are memorable for consumers.

## Significance

In the creation of new brand names companies often have two main goals: to create a name that conveys some characteristic or information about the product and is easily remembered by consumers. The use of sound symbolism to create names that “fit” well with certain products has been a topic of interest in marketing research (Klink, 2000, 2009). However, this research has been limited by stimulus sets with definite categorical boundaries (e.g., Aveyard, 2012; Westbury, 2005) which do not accurately reflect the variability inherent in naturally occurring language and the common use of a forced choice task to obtain size judgments (i.e., large or small; e.g., Klink, 2000, 2009; Sapir, 1929). To our knowledge, the extent to which sound symbolic properties might affect the memorability of potential brand names has yet to be directly examined. In the present study, we extended upon the results of this previous work by using a set of novel nonword stimuli with considerable variability and a more continuous size scale for participant size judgment. Additionally, we examined whether sound symbolism could enhance memory for nonword-definition pairs. Brand names that adhered to sound symbolism “rules” were more memorable. This work supports the use of sound symbolism in the creation of effective brand names and provides tools for those creating these names in a competitive marketing world.

## Background

A single word can be the most powerful tool of any author, poet, or advertiser. Ultimately, words are a combination of sounds or phonemes within a particular language. In traditional linguistic theory, the morpheme is the unit in language that conveys meaning, and individual phonemes serve a primarily contrastive function (i.e., they discriminate between similar morphemes). Thus, the phonemes a morpheme is composed of are arbitrarily related to one another (Ohala, Hinton, & Nichols, 1994). For example, the/ʃ/[sh] sound can be found in many words (*sheet, ship, show, sharp*), but none of these words share meaning. One notable exception to this general rule is in the case of onomatopoeia, where the sound of a word conveys its real-world referent, as in *zoom* or *buzz*. Although onomatopoeic words are relatively rare, especially in Anglo-Saxon languages, they suggest that in some cases the relationship between sound and meaning may not always be arbitrary. Such an assumption challenges traditional notions that the morpheme is the smallest unit that conveys meaning and suggests that, in some cases, individual phonemes or phonemic combinations might communicate meaning directly.

According to sound symbolism theory, sounds (e.g., phonemes, phonetic features) are directly involved in conveying meaning (Nuckolls, 1999; Sapir, 1929). Sound symbolic effects likely occur on a continuum and vary across languages in their prevalence. At one extreme, onomatopoeia represents the most direct sound to meaning link. In Japanese, mimetics are not only fairly common, but constitute an open class, and other language groups include ideophones and expressives, which are similar in nature (see Imai, Kita, Nagumo, & Okada, 2008; Nuckolls, 1999). In Indo-European languages, a relatively large class of sound symbolic elements are phonesthemes, sound sequences, or clusters that occur across a number of words that have similar semantic properties (e.g., *glimmer, glisten, glow* refer to steady light and *flicker, flash, flare* refer to moving light; Bergen, 2004; Parault & Parkinson, 2008).

The origins of phonetic sound symbolism mappings are intertwined with the origins of all spoken language. According to the cross-modal theory (Thompson & Estes, 2011), sound symbolism developed systematically based on the physical properties of referents, the speech “gestures” found in a word (i.e., the shape created with the mouth when pronouncing a phoneme, such as the rounded mouth shape of/o/), and the frequency/pitch of the phonemes found in the word or emitted by the referent. This suggests that sound symbolism mappings should be similar across languages because the mappings would be directly based on referents. In fact, in 90% of languages, small size is symbolized by high front vowels such as*/i/*(Ultan, 1978). Additionally, almost all languages provide evidence supporting sound symbolism effects (Ciccotosto, 1991) such as phonesthemes. However, there are some exceptions where mappings between languages differ, such as the front vowels that indicate smallness in English indicating largeness in Bahnar (Diffloth, 1994).

Sound symbolism research often uses nonwords, which are pronounceable letter strings (e.g., *golon, anibi*). In typical studies, participants report the properties they associate with nonwords, which contain vowels and consonants that can represent particular physical characteristics such as shape or size. For example, *takete* and *kiki* are typically associated with angular shapes, whereas *maluma* is associated with rounded shapes (Köhler, 1929). Different classes of sounds (e.g., plosives, fricatives) are associated with different perceptual properties (Fort, Martin, & Peperkamp, 2014; Nielsen & Rendall, 2011).

One of the most researched sound symbolic effects is that of size or magnitude symbolism. In an early study, Sapir (1929) presented participants pairings of three letter nonwords following the pattern of consonant-vowel-consonant (CVC), only differing by the middle vowel. One nonword contained a back vowel sound and the other a front vowel sound. Front vowels are produced when the tongue’s highest point is toward the front of the mouth during sound production such as the vowel phonemes/ɪ/and/e/(e.g., the [i] in *bit* and the [ee] in *bee*)*.* Back vowels are produced when the highest point of the tongue is toward the back of the mouth such as in the vowel phonemes/o/(e.g., the [o] in *boat*) and/u/(e.g., the [oo] in *boot*; Klink, 2009). Sapir (1929) tested which of the artificial words conveyed “smallness” or “largeness” by giving participants a referent such as a table. Generally, back vowel nonwords were associated with largeness (i.e., the large table) and front vowel nonwords with smallness (i.e., the small table). Over 80% of participants were in agreement across a large number of nonword pairs. Sapir found similar results in children, university students, American adults, and Chinese speakers, confirming the generality of the effect.

More recently, Klink (2000) reported that front vowels were associated with lighter colors and weight, thinness, weakness, softness, coldness, bitterness, and femininity compared to back vowels, which were associated with largeness and the opposite of all the adjectives mentioned in reference to front vowels. Klink (2000) also manipulated consonants, specifically stops vs. fricatives. Stops and fricatives describe the position of articulators when pronouncing these consonants. Stops have complete closure of the articulators before releasing the airstream from the mouth, producing a hard stop like that heard in the phonemes/p/,/t/,/b/,/g/,/d/, and/k/(e.g., [p] in *pill*, [t] in *till*, [b] in *bill*, [g] in *gill*, [d] in *dill*, and [k] in *kill*). Fricatives are characterized by a less sudden stop of the airstream leaving the mouth during articulation, as in the phonemes/f/,/s/,/v/, and/z/(e.g., [f] in *feel*, [s] in *seal*, [v] in *veal*, [z] in *zeal*). Nonwords containing fricatives were perceived as smaller, faster, lighter, sharper, softer, and more feminine than stops (Klink, 2000). Thus, both consonants and vowels seem to be associated with distinct physical characteristics.

Brand names can be considered an applied example of sound symbolic properties of phonemes. Brand names often are developed to intentionally convey a feature or characteristic of the product they represent, such as *Viagra*, in which the letter V is associated with ideas of energy (Begley, 2002). Applications of the communicative properties of speech sounds have been conducted in the area of marketing of brand names. It is likely that sound symbolism is an essential part of brand name development due to the meaning it can contribute to nonwords, which can be strongly associated with product or brand categories and more memorable than word brand names (Coane, Monahan, & Termonen, 2015). The interplay of meaning and the importance of speech sounds are clear in marketing contexts. For example, semantic appositeness or appropriateness (i.e., the extent to which a name conveys information about the product; e.g., the air freshener brand *Febreze* being a combination of the words *fresh* and *breeze*; Lowrey, Shrum, & Dubitsky, 2003) and initial plosives were the two most common characteristics of the top 200 brand names listed in the annual *Marketing and Media Decisions* from 1971 to 1985 (Vanden Bergh, Adler, & Oliver, 1987). To our knowledge, the extent to which sound symbolic properties might affect the memorability of potential brand names has yet to be directly examined. If characteristics of a brand or product name in some way facilitate recognition by making it easier to remember what the product is, this could support the development of meaningful and memorable product labels as well as provide further insights into how the meanings conveyed by sounds influence linguistic processing and memory.

Related work suggests that sound symbolism effects can influence word learning, even when these words are foreign to the learner. When learning the meanings of obsolete English words, participants were better at guessing and recognizing words that included sound symbolic phonemic clusters compared to non-sound symbolic words (Parault & Schwanenflugel, 2006). For example, *scriek* refers to a cry or sound and *bauch* means indifferent or insipid. Similar results were found with middle school children when asked to guess or select definitions for sound symbolic stimuli vs. non-sound symbolic stimuli both in isolation and embedded in a sentence context (Parault & Parkinson, 2008). Neither initial sound associations nor mediated associations from the target to a definition (e.g., *scriek → shriek → cry*) seemed to solely drive the effect (Nygaard, Cook, & Namy, 2009; Parault & Schwanenflugel, 2006). Furthermore, children learned words that were sound symbolically related to their definitions faster and easier than arbitrarily related words and definitions (Berko-Gleason, 2005). Imai et al. (2008, 2015) have found sound symbolism facilitation in word learning in children as young as 14 and 25 months old. Cross-linguistic support was found in Nygaard et al.’s (2009) study, in which native English speakers learned unfamiliar Japanese words. Together, these studies suggests that sound symbolism is one of many tools learners have when faced with the task of learning new words. These studies used foreign language or obscure words, which are similar to nonwords in that they do not have a pre-existing semantic representation. This is often the case in marketing and branding: a novel word or nonword is created for the purpose of uniquely identifying a product and thus its meaning must be learned.

In the present series of studies, we examined how sound symbolic nonwords were processed and remembered. We focused on size as a property because of the clearly identified sound categories associated with size (Klink, 2000, 2009; Sapir, 1929). Many earlier studies used limited stimulus sets with definite categorical boundaries, such that nonwords were unambiguously large or small in terms of the component phonemes (e.g., Aveyard, 2012; Westbury, 2005). Such stimuli do not accurately reflect the variability inherent in naturally occurring language, where additional constraints might be in place. For example, initial plosives and semantic appositeness may facilitate recall (Lowrey et al., 2003); however, these features may be incompatible with the size of the product being marketed. We developed a large set of stimuli with more intra-category variability to mimic naturally occurring words and to reduce stimulus-specific effects. Another notable difference is that most prior studies employed a forced choice task to obtain size judgments (i.e., large or small; e.g., Klink, 2000, 2009; Sapir, 1929), whereas we used a variety of less constraining measures of perceived size. Specifically, we asked participants to rate size of a set of nonwords using a continuous scale, to provide open-ended descriptions of a possible referent to the nonword, and to provide free associations to the nonwords.

Based on prior research (e.g., Klink, 2000, 2009; Sapir, 1929), nonwords containing front vowels and fricatives should be rated as smaller than nonwords containing back vowels and plosives. Similarly, nonwords containing “small” sounds should be described as, and associated with, small objects if sound symbolic phonemes activate broader concepts of size. Such findings would suggest that, in the absence of a pre-existing mental representation, participants rely on sound symbolic qualities of stimuli. An alternative hypothesis is that sound symbolism effects in nonwords might be mediated by their word neighbors. Thus, a nonword like *blomp* might be considered large because of its orthographic neighbor, *blimp.* Free association data, in which participants generated one-word responses to the nonwords, allowed us to examine the influence of related concepts on judgments of size.

Finally, we examined whether associative learning processes are influenced by sound symbolism. Participants studied nonwords with either sound symbolism congruent (e.g., a small nonword paired with a definition of a small object) or incongruent definitions (e.g., a small nonword paired with a large object) and then completed a recall task. Similarity and relatedness exert robust effects on memory, with related or semantically similar items showing a recall and recognition advantage relative to unrelated items (e.g., Cofer, Bruce, & Reicher, 1966; Kintsch, 1968; Roediger & McDermott, 1995). If sounds associated with smallness or largeness provide access to size-related information, this should facilitate learning of congruent pairs relative to incongruent items because of the greater similarity between the nonword and the definition. As such, this should result in a memory advantage for congruent over incongruent items, consistent with prior studies (e.g., Parault & Parkinson, 2008; Parault & Schwanenflugel, 2006) reporting advantages for sound symbolic word learning.

## Experiment 1

Two studies were conducted to develop nonwords and to assess the extent to which they were associated with concepts of largeness or smallness. In Experiment 1A, participants rated 100 nonwords for size using a continuous scale that was congruent with a standard number line (i.e., increasing from left to right) without visible numerical labels. In this way, participants were discouraged from repeating scale ratings between items and responded in a more nuanced way (i.e., by not forcing participants to classify something as large or small, we tested the extent of sound symbolic effects). In Experiment 1B, different participants provided open-ended definitions to the same 100 nonwords. The definitions were coded in terms of the size of the referents in the definitions.

### Experiment 1A

#### Participants

Participants were 15 undergraduate students at a liberal arts college and 335 people recruited online through Amazon Mechanical Turk. Mechanical Turk participants provide data as reliable as those obtained via traditional methods (Buhrmester, Kwang, & Gosling, 2011). Undergraduate students were offered partial course credit for their participation. Participants recruited through Mechanical Turk were compensated $0.50. Mean age was 36.66 years (*SD* = 12.78; range = 18–75) and participants’ mean reported education level was 15.30 years (*SD* = 2.55; range = 10–25). Participation took approximately 5 min. Seven (2%) participants were not native speakers of English; all indicated fluency (Mechanical Turk participation was limited to the USA).

#### Materials

The stimuli were 100 nonwords (see Appendix 1) selected from the English Lexicon Project (http://elexicon.wustl.edu; Balota et al., 2007). All nonwords had an orthographic neighborhood between 1 and 3 (*M* = 1.16) and were five letters long. Utilizing the sound symbolism phoneme distinctions identified in previous research (Klink, 2000; Sapir, 1929), we selected nonwords that mostly contained consonants and vowels associated with largeness (i.e., the phonemes/p/,/t/,/b/,/d/,/g/,/k/,/o/and/u/) or smallness (i.e., the phonemes/f/,/s/,/v/,/z/,/l/,/r/,/ɪ/, and/e/)*.* All nonwords contained at least one vowel and one consonant from their respective size category and, whenever possible, the other included letters came from this size category or were “neutral” letters (not specified by previous research to have any strong sound symbolism effects for size). For the sake of brevity, we refer to nonwords composed primarily of sounds associated with largeness as “large nonwords” and those composed of sounds primarily associated with smallness as “small nonwords.”

A breakdown of the characteristics of the stimuli used is presented in Table 1. The main consonantal manipulation—plosive vs. fricative—was clearly distinguishing the two categories of nonwords, with large nonwords including a greater proportion of plosives than small nonwords, and small nonwords including more fricatives than large nonwords. Similarly, the proportions of vowels also differed across large and small categories in line with the distinctions made in previous research. Although stimuli were selected primarily on the basis of the plosive/fricative distinction identified in earlier work (e.g., Klink, 2000, 2009), other phonemes were included to ensure the stimuli were not overly similar within each classification of intended size. In other words, we did not want the artificial categories to be completely confounded with specific phonetic classes. Clearly, this would decrease any effects of sound symbolism (because the categories would overlap more) but would increase the ecological validity of the stimuli, because including “noise” in the categories would, if anything, decrease the likelihood of detecting sound symbolic effects, and would thus be indicative of the robustness of the effect.

**Table 1:** Phonetic characteristics of nonwords used in Experiment 1

	Large nonwords		Small nonwords		
	Mean	SD	Mean	SD	*p* value
Number of vowels	1.76	0.62	2.10	0.79	0.02
Proportion “large” vowels	0.85	0.22	0.04	0.16	<0.001
Proportion “small” vowels	0.03	0.11	0.54	0.36	<0.001
Number of consonants	2.88	0.66	2.60	0.70	0.04
Total voiced consonants	0.63	0.29	0.62	0.29	0.86
Total unvoiced consonants	0.37	0.29	0.38	0.29	0.86
Total plosives	0.53	0.22	0.17	0.24	<0.001
Total fricatives	0.12	0.19	0.45	0.31	<0.001
Total affricates	0.03	0.10	0.01	0.05	0.09
Total nasals	0.12	0.21	0.07	0.17	0.20
Total approximants	0.20	0.20	0.27	0.24	0.11
Total consonant clusters	0.23	0.23	0.15	0.17	0.05
Total vowel clusters	0.11	0.20	0.08	0.21	0.47
Onset position voiced consonants	0.54	0.50	0.32	0.47	0.03
Onset position unvoiced consonants	0.32	0.47	0.24	0.43	0.38
Onset position plosives	0.56	0.50	0.08	0.27	<0.001
Onset position fricatives	0.20	0.40	0.34	0.48	0.12
Onset position affricates	0.06	0.24	0.00		0.08
Onset position nasals	0.06	0.24	0.06	0.24	>0.99
Onset position approximants	0.00		0.08	0.27	0.04
Onset position consonant clusters	0.36	0.49	0.20	0.40	0.08
Onset position vowel clusters	0.00		0.00		n.a.

*Note*: consonants were defined at the phonemic not orthographic level (e.g.,/ch/was counted as a single consonant) and dipthongs were counted as two vowels. Double letters were counted as a single occurrence. In calculating front and back vowels, final-position *e* (i.e., the silent e) was not counted. *n.a.* not applicable

Participants saw five randomly selected nonwords from each size category. The experiment was completed via Qualtrics online survey software (2015; Qualtrics, Provo, UT, USA; http://www.qualtrics.com). Slider bar responses were on a 100-point scale, with 0 being the smallest and 100 being the largest. These numerical scale points were invisible to participants.

#### Procedure

In all experiments, participants gave informed consent prior to participation and provided information about their age, gender, and education level. Next, participants were told they would be shown 10 brand names being considered for new products. For each brand name, participants were instructed to use a slider bar with one end labeled *small* and one end labeled *large* to rate how small or large the brand name felt to them. The slider bar had no numerical markings on it to encourage a unique response to each item. Participants could not easily recall and repeat a rating given to a previously seen item. It was emphasized that there were no right or wrong answers and that the experimenters simply were interested in how people react to these brand names. All participants were debriefed after completion of the study.

#### Results

Because different participants rated different subsets of nonwords in this and the following study, the primary analyses are at the item level, averaged across participants (approximately 30 participants responded to each item). Participants rated nonwords consisting of “small” sounding letters (*M =* 38.46, *SD* = 6.73) as smaller than nonwords consisting of “large” sounding letters (*M* = 50.07, *SD* = 7.23), *t*(98) = 8.31, *p* < 0.001, Cohen’s *d* = 1.66. There was substantial overlap in the distributions (small nonwords range = 20.83–54.56; large nonwords range = 38.43–68.89), suggesting the effect is quite variable, although the upper and lower bounds of the two distributions are consistent with predictions.

To examine whether specific phonemic characteristics (e.g., plosivity, voicing) predicted rated size, we conducted some correlational analyses using the phonemic characteristics listed in Table 1. A positive correlation indicates that the more a specific characteristic is present in the nonwords, the larger the rated size, whereas a negative correlation would suggest that as the presence of a specific feature increases, rated size decreases. Thus, we would expect that presence of plosives would be positively related to size, and fricatives would be negatively related to size. In fact, this prediction was supported by the analyses. When nonwords included more plosive consonants the size increased (*r* = 0.41, *p* < 0.001), and when nonwords included more fricatives the rated size decreased (*r* = –0.37, *p* < 0.001). None of the remaining consonantal features (i.e., voicing, number of nasals, affricates, or approximants) predicted rated size (all *p* > 0.40). Turning to the vowels, as the number of back vowels increased so did perceived size (*r* = 0.63, *p* < 0.001), whereas as the number of front vowels increased the perceived size decreased (*r* = –0.62, *p* < 0.001).

Because the stimuli were developed to intentionally include plosives or fricatives and back or front vowels, respectively, different phonological properties were confounded. The correlation between fricatives and front vowels was 0.36 and the correlation between plosives and back vowels was 0.50 (fricatives and back vowels were correlated at –0.51 and plosives and front vowels were correlated at –0.48). A multiple regression analysis was performed entering the four correlated properties (plosives, fricatives, front vowels, and back vowels). Only vowel properties predicted size: back vowels were positively associated with rated size (*b* = 6.98, *t*(95) = 2.97, *p* = 0.004) and front vowels were negatively associated with size (*b* = –8.10, *t*(95) = –3.0, *p* = 0.003). The regression predicted 0.44 of the variance, although these results should be interpreted with some caution given the strong correlations between predictors. These analyses suggest that the primary factor influencing perceived size in the present stimulus set is the front/back distinction among vowels. Of course, it is important to note that the non-significant effects of some other phonemic characteristics might have been due to the small number of occurrences of specific features. Overall, the stimuli used in the present study are consistent with the distinctions made by Klink and others (e.g., Klink, 2009) as well as supporting the hypothesis that magnitude sound symbolism is not an all-or-none phenomenon but likely occurs on a graded continuum (cf. Thompson & Estes, 2011).

### Experiment 1B

#### Participants

Participants were 77 undergraduate students at a private liberal arts college and 259 people recruited online through Amazon Mechanical Turk. Undergraduate students were offered partial course credit for their participation. Participants recruited through Mechanical Turk were compensated $0.65. Mean age was 33.32 years (*SD* = 13.58; range = 18–72). Mean years of education were 14.88 years (*SD* = 2.50; range = 10–24). Thirty-one participants failed to complete the task; thus, analyses are based on data from 305 participants. Three participants (0.09%) indicated English was not their first language. Participation in the study took approximately 10 min.

#### Materials

Stimuli were the same as described in Experiment 1A.

#### Procedure

Participants were informed they would be shown 10 fictitious brand names for new products and were asked to type a sentence-long description of the object they felt the brand name should represent. Participants were asked to make this description about the object’s physical appearance and size rather than any other qualities. It was emphasized that there were no right or wrong answers and that the experimenters were simply interested in how people responded to these brand names. A written debriefing form was presented after completion of the study.

#### Results

Responses to the open-ended definitions were scored for objective size using a shoebox as a referent point. The shoebox was selected as a referent because of its common usage in many cognitive tasks, in particular semantic categorization tasks (e.g., Dobbins, Schnyer, Verfaellie, & Schacter, 2004; Schacter, Wig, & Stevens, 2007; Uncapher, Otten, & Rugg, 2006). Anything smaller than a shoebox was coded as small, and anything larger than a shoebox was coded as large. In addition to coding the object listed in the definition in terms of size, the presence of the words *small* or *large* (or synonyms, such as *handheld*, *bulky*) was scored. A final coding category was for items for which no size could be determined (e.g., “something European” or “a position”) or when the size was ambiguous (e.g., car part) or the size given was medium. The following guidelines were created for use when coding definitions. For example, definitions that used the word *small* to describe something relatively large in comparison to a shoebox (e.g., *a small wheelbarrow*) were coded as large objects with a small size descriptor. Raters looked at photos of objects online (doing a Google image search) when determining their size. For some object categories, specific instructions were developed. Fabric items that could be folded to fit inside a shoebox (e.g., shirts, scarfs) were coded as small, bulky items that could not fold to fit inside a shoebox (e.g., duvets, winter coats) were coded as large. Appliances were considered large if they were bigger than a microwave (e.g., oven, fridge, deep fryer) and smaller if they were equal in size or smaller than a microwave (e.g., toaster, blender, coffee pot).^1^ Tool sizes were often determined by searching on-line for the participant-provided description of the tool and looking at the resulting pictures or based on any size-related adjectives provided.

Two raters, one blind to the purpose of the experiment, coded the data separately. Initial correlation between scorers was at 0.85 or higher on all coding elements; discrepancies were resolved by the lead author.

The proportion of given responses across nonword size were analyzed using independent samples *t* tests (see Table 2 for means). Overall, small nonwords were described as small objects more than large nonwords (*t*(98) = 2.18, *p* = 0.03, Cohen’s *d* = 0.44). The proportion of times the word *small* was used in the definitions did not differ as a function of nonword size, although, numerically, small nonwords did include the word small more than large nonwords (*p* = 0.26). Conversely, large objects were included in the definitions more for large nonwords than for small nonwords (*t*(98) = 2.38, *p* = 0.02, Cohen’s *d* = 0.48) and the word *large* was more frequently included in definitions of large nonwords than small nonwords (*t*(98) = 2.62, *p* = 0.01, Cohen’s *d* = 0.53). There was no difference in the proportion of times small and large words could not be classified as sized objects (*p* = 0.82). Given the overall low response rates in most categories, additional analyses examining individual contributions of the key phonological features were only performed on the proportion of “small object” responses. The number of fricatives emerged as a modest predictor of small object responses (*b* = 0.04, *t*(95) = 1.73, *p* = 0.09), and the number of plosives was negatively associated, albeit weakly, with small object responses (*b* = –0.032, *t*(95) = 1.56, *p* = 0.12). Vowel counts were unrelated to small object responses (both *p* > 0.53). Thus, in contrast to the size rating task, in the definition task, consonantal properties appeared to have a larger influence on perceived size than vowel properties.

**Table 2:** Mean number of nonword participant-generated definitions coded for small and large object size and small and large adjective use in Experiment 1B (standard errors in parentheses)

	Nonword size		
	Small	Large	*p* value
Defined as small object	0.82 (0.02)	0.76 (0.02)	0.03
Word *small* in definition	0.24 (0.01)	0.22 (0.01)	0.26
Defined as large object	0.14 (0.01)	0.19 (0.02)	0.02
Word *large* in definition	0.09 (0.01)	0.13 (0.01)	0.01
No size given/ambiguous	0.04 (0.01)	0.04 (0.01)	0.82

Overall, most definitions referred to small objects. The use of the term *product* in the instructions may have affected the size ratings and the size descriptions because *product* might imply an object that fits onto a store shelf. This may have led the participants of Experiments 1A and 1B to believe their responses needed to be such objects, resulting in a bias towards small responses.

In addition to the referent-based scoring at the item level, we conducted analyses by participant by assigning a relative size score to each definition. Each participant’s definitions were ranked from small to large on a 1–10 scale, where the smallest items were given a 1 and largest items a 10. Items at or below the midpoint were considered small and those 6 and above were considered large. This coding scheme also allowed us to address the concern about the small object response bias. A power analysis using the lowest effect size from Experiment 1A (*d* = 0.44) indicated 70 participants were needed to detect a two-tailed effect with 0.95 power (Faul, Erdfelder, Lang, & Buchner, 2007). We selected approximately one-third of the data (i.e., 106 participants); 18 participants provided responses that could not be reliably coded for size (e.g., by referring to color, shape, or speed properties for a number of the definitions). Thus, data from 88 participants were included in the following analyses. Two coders, who were blind to the purpose of the study, ranked the definitions provided by each participant. Initial correlation between raters was 0.81; discrepancies were resolved through discussion until 100% agreement was achieved. Consistent with the previous analyses, participants rated small nonwords smaller (*M* = 5.11, *SD* = 0.87), on average, than large nonwords (*M* = 5.89, *SD* = 0.87) (*t*(87) = 4.15, *p* < 0.001, Cohen’s *d* = 0.63). Thus, even when the definitions were scored using relative, as opposed to absolute, criteria, the same pattern of results emerged.

### Discussion

Experiments 1A and 1B suggested that the 100 novel nonword stimuli could elicit sound symbolism effects. This was especially true in Experiment 1A, in which participants rated the “small” nonwords significantly smaller than the “large” nonwords and vice versa. It is important to note, however, that the magnitude of this difference was not as large as those seen in previous research (e.g., Klink, 2000), who reported a highly significant difference for the majority of tested word pairs (i.e., *p* < 0.001). This might be due to the fact that the slider bar design used in Experiment 1A is a more sensitive measure and allowed participants to make more nuanced decisions. Alternatively, the nonwords in the present study might be more variable or less extreme in terms of sound symbolic mappings.

Sound symbolism effects, albeit smaller, were also observed in Experiment 1B. These results suggest that sound symbolism effects can be captured, even when participants are allowed to freely associate definitions for nonwords. The size of the objects defined varied systematically with nonword size when the responses were scored using an objective referent, such as a shoebox, and when they were scored by ranking each participant’s responses from small to large. Although these effects were subtler than those found in forced choice tasks, the results suggest that these effects can be spontaneously produced in the manner many marketers hope to achieve when they send a new nonword brand name into the market.

## Experiment 2

Experiment 2 utilized the Experiment 1 nonwords in a memory task. The nonwords were paired with three-word definitions selected from the participant-generated definitions from Experiment 1B. The experiment was conducted twice, with nonword-definition congruency, described below, as a between-subjects factor (Experiment 2A) and as a within-subjects factor (Experiment 2B).

### Participants

All participants were recruited via Amazon Mechanical Turk and tested online. Sixty individuals were tested in the between-subjects design (30 in each condition) and 74 in the within-subjects design.^2^ All participants were compensated $1.00 for their participation. In the between-subjects design, mean age was 38.89 years (*SD* = 12.95; range = 19–77) and mean years of education were 15.87 (*SD* = 2.60; range = 12–27). Two (3.3%) participants were non-native speakers of English. In the within-subjects design, mean age was 37.29 years (*SD* = 11.44; range = 20–77) and mean years of education were 15.03 (*SD* = 2.31; range = 12–23); one participant (1.3%) was not a native English speaker. Participation took approximately 20 min.

### Materials

The stimuli were 28 nonwords. We selected a subset of the stimuli from Experiment 1 to create a memory test that would not exceed participants’ retention ability. To ensure that our small and large categories reflected our intended manipulation, we selected the 14 nonwords rated as smallest (*M* = 31.17, *SD* = 4.77) and 14 nonwords rated as largest (*M* = 58.77, *SD* = 5.43) in Experiment 1A. The characteristics of the stimuli used in Experiments 2–4 are presented in Table 3. An important feature of these stimuli is that they not only map onto existing phonological and phonemic mappings identified in the literature, but they also were rated by participants as large or small.

**Table 3:** Phonetic characteristics of nonwords used in Experiments 2-4

	Large nonwords		Small nonwords		
	Mean	SD	Mean	SD	*p* value
Number of vowels	1.64	0.50	2.07	0.92	0.14
Proportion “large” vowels	0.86	0.23	0.00	0.00	<0.001
Proportion “small” vowels	0.00	0.00	0.74	0.29	<0.001
Number of consonants	3.07	0.62	2.50	0.76	<0.001
Proportion voiced consonants	0.68	0.35	0.60	0.32	0.54
Proportion unvoiced consonants	0.32	0.35	0.40	0.32	0.54
Proportion plosives	0.46	0.13	0.20	0.22	0.001
Proportion fricatives	0.14	0.20	0.41	0.37	0.03
Proportion affricates	0.02	0.09	0.02	0.09	>0.99
Proportion nasals	0.12	0.18	0.12	0.20	0.94
Proportion approximants	0.24	0.17	0.25	0.20	0.93
Proportion consonant clusters	0.32	0.24	0.14	0.16	0.03
Proportion vowel clusters	0.07	0.18	0.07	0.18	>0.99
Onset position voiced consonants	0.27	0.16	0.09	0.15	0.006
Onset position unvoiced consonants	0.05	0.12	0.08	0.17	0.53
Onset position plosives	0.20	0.16	0.02	0.09	0.001
Onset position fricatives	0.09	0.19	0.10	0.17	0.93
Onset position affricates	0.02	0.09	0.00	0.00	0.33
Onset position nasals	0.00	0.00	0.02	0.09	0.33
Onset position approximants	0.00	0.00	0.02	0.09	0.33
Onset position consonant clusters	0.15	0.16	0.07	0.14	0.18
Onset position vowel clusters	0.00	0.00	0.00	0.00	n/a

*Note*: consonants were defined at the phonemic not orthographic level (e.g.,/ch/was counted as a single consonant). *n.a.* not applicable

Each nonword was paired with a shortened version of a participant-generated definition from Experiment 1B. Definitions were created by examining the definitions generated by participants in Experiment 1B and noting recurring themes in the definitions (e.g., several participants indicated that *eevie* was a small electronic device of some sort). Each definition consisted of three words: a size adjective, a non-size adjective, and a noun (e.g., *Blomp: Large soft chair*). The size adjectives included adjectives which were not directly size descriptive, but instead suggestive of a size (e.g., *portable* suggests smallness) to reduce repetition. The definitions provided by participants varied widely, with many responses only being given by one or two participants, but, on average, across all 28 nonword-definition sets the adjective component (or a near synonym) was given by 11% of participants (range 0-60%) and the noun component was given by 16% of respondents (range 3-67%). Definitions were also selected based on plausibility and clarity and the ease with which a three-word definition could convey a distinct idea. Sample definitions were: *small air spray*, *large sports car*, and *portable smooth lotion*. Across nonword size categories, the words within the definitions were matched on length, the log transformed frequency of the words from the Hyperspace Analog to Language (HAL; Burgess & Livesay, 1998), subtitle frequency (Brysbaert & New, 2009), orthographic neighborhood (Coltheart, Davelaar, Jonasson, & Besner, 1977), mean response time and mean accuracy in lexical decision from the ELP (Balota et al., 2007), and their co-occurrence within the English language based on latent semantic analysis (LSA; Landauer, Foltz, & Laham, 1998).

Nonword-definition pairs were congruent or incongruent. Seven nonwords of each size were paired with size-congruent definitions and seven with size-incongruent definitions. Congruency was given by the match between the implied size of the nonword (large or small) and by the size adjective in the definition (large or small). In the congruent condition, nonwords appeared with a sound symbolism-size congruent definition (e.g., *Zrizz: small child zipper, Blomp: large soft chair*). In the incongruent condition, nonwords were re-paired with definitions for nonwords of the other size (e.g., *Zrizz: large soft chair, Blomp: small child zipper*). Each nonword was presented with a congruent or incongruent definition across participants for counterbalancing purposes.

Because the definitions were based on participant responses, it is possible that characteristics of the adjective and noun component reflected effects of sound symbolism or other associations not directly related to size. Congruent definitions possibly reflected some influence of onomatopoeia (e.g., *zrizz* being associated with the sound of a zipper), object-sound associations (e.g., *thuap* might reflect the sound a fly swatter makes), and other indirect relationships between phonology and meaning. Factors such as onomatopoeia or sound associations at the level of the nonword (as in *thuap*) are likely to affect the ease with which a nonword and definition are associated and remembered. This would result in a mnemonic advantage for the non-size components of the definition as well as the size component. To examine this, we report analyses that discriminate between memory for the entire definition and its components and, in Experiment 3, attempt to eliminate all forms of congruency other than the size information.

### Procedure

Participants studied 28 nonwords and their definitions for an unspecified memory task. Each nonword-definition pair remained on screen for 8 s before the next pair was presented. The pairs appeared in random order for each participant. After the study phase, participants were given a cued recall task. Each of the 28 nonwords appeared on screen in random order and the participant typed the definition the nonword had been paired with in the study phase. Participants were instructed to write as much of the three-word definition they could remember. If they could not remember the definition at all, they were asked to leave the textbox blank. All participants were debriefed after completion of the study.

Scoring of participant responses was based on a 0–3 point system. If no answer or a completely incorrect answer was given the participant received a 0, if 1 out of the 3 words (or a near synonym) was remembered the participant received 1 point, and so forth. Noun synonyms were counted as correct (e.g., *cleaner* instead of *detergent*). Size adjectives were scored both liberally (i.e., synonyms such as tiny or small were coded as correct) and using stringent criteria such that size adjectives were coded as correct only if they were reproduced verbatim. If the effect was driven by the same implicit associations that emerged in the size rating and definition tasks, participants might have been biased to guess that an object was small or large. The more conservative scoring would reduce the effects of such biases.^3^ For the sake of brevity, we only report the latter analyses (results were largely consistent in the more liberal analyses).

### Results

Analyses by subjects (*F*_*1*_) and items (*F*_*2*_) are reported. The former provide insight into whether, at the participant level, sound symbolic effects can be detected in a memory task. For the latter, two complementary analyses are reported. First, analyses by items using the experimenter-defined category of large vs. small nonwords are reported. Second, ANCOVAs and regression analyses were conducted using mean-centered frequency of phonological features instead of the subjective classification of size to explore the relative influence of the sound-symbolic phonological properties. All effects were significant at the *p* < 0.01 level unless otherwise indicated, and Bonferroni corrections were applied to pairwise comparisons where relevant.

### Experiment 2A – between-subjects design

**Fig. 1:**
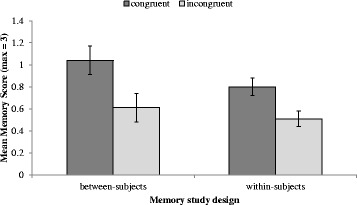
Mean memory score out of three possible points as a function of congruency in Experiments 2A (between-subjects) and 2B (within-subjects). Error bars reflect the standard error of the mean

Due to a programming error, one stimulus (*afain*, a large nonword) was missing in six cases in the incongruent condition. Total recall scores (out of 3) were submitted to a 2 (nonword size) × 2 (congruency) mixed ANOVA, in which size was a within-subjects factor and congruency a between-subjects factor in the participant analyses and vice versa in the item analyses (see Fig. 1 for overall memory performance scores). The analysis on total correct recall revealed a significant main effect of congruency (*F*_*1*_(1, 58) = 4.74, *p* = 0.03, η^2^_p_ = 0.08, *F*_*2*_(1, 26) = 62.96, η^2^_p_ = 0.71), with congruent items (*M* = 0.84, *SEM* = 0.12) recalled more than incongruent items (*M* = 0.46, *SEM* = 0.12). Neither the effect of nonword size nor the interaction were significant (*F*_*1*_(1, 58) = 0.58, *p* = 0.46, *F*_*2*_(1, 26) = 0.19, *p* = 0.67; and *F*_*1*_(1, 58) = 0.44, *p* = 0.51, *F*_*2*_(1, 26) = 0.21, *p* = 0.65, respectively). Turning to the analyses on memory for the size component only, a significant interaction emerged (*F*_*1*_(1, 58) = 7.43, η^2^_p_ = 0.11), which reflected a significant congruency effect for small nonwords (*M*_*congruent*_ = 0.32, *M*_*incongruent*_ = 0.17, *t*[58] = 2.09, *p* = 0.04, Cohen’s *d* = 0.54) but none for large nonwords (*t*(58) = 0.61, *p* = 0.54). Neither the effect of congruency (*F*_*1*_(1, 58) = 2.0, *p* = 0.16) nor the main effect of nonword size (*F*_*1*_(1, 58) = 0.45, *p* = 0.50) were significant. The item analyses revealed a significant effect of congruency (*F*_*2*_(1, 26) = 11.75, η^2^_p_ = 0.31) and a marginally significant interaction (*F*_*2*_(1, 26) = 3.59, *p* = 0.069, η^2^_p_ = 0.12), and no effect of nonword size (*F*_*2*_ (1, 26) = 0.24, *p* = 0.63). Finally, when we examined memory for the adjective and noun components (calculated as the difference between total score and verbatim memory score for size), a significant congruency effect was present (*F*_*1*_(1, 58) = 6.37, *p* = 0.014, η^2^_p_ = 0.10 (*M*_*congruent*_ = 0.56, *M*_*incongruent*_ = 0.28), *F*_*2*_(1, 26) = 65.99, η^2^_p_ = 0.72), and no effect of nonword size and no interaction (*F*_*1*_(1, 58) = 0.26, *p* = 0.61, *F*_*2*_(1, 26) = 0.10, *p* = 0.75 and *F*_*1*_(1, 58) = 0.99, *p* = 0.32, *F*_*2*_(1, 26) = 0.88, *p* = 0.36, respectively). In summary, overall memory was enhanced for congruent items relative to incongruent items, although other, semantically-mediated associations, do contribute to the congruency effect.

For the secondary item analyses, memory scores for “large” definitions and “small” definitions were treated as a within-items factor and the four key phonological features (number of plosives, number of fricatives, number of front vowels, number of back vowels) were entered as covariates. In other words, we did not code responses as congruent/incongruent as in the participant analyses and did not use the experimenter-defined small/large classification, but focused on whether an interaction between the size implied by the definition and the phonological properties emerged. The interaction, then, would be analogous to a congruency effect (e.g., the more large vowels in a nonword, the better a large definition is recalled). For overall memory performance, the number of large vowels and definition size interacted (*F*(1, 23) = 6.53, *p* = 0.02, η^2^_p_ = 0.22). Follow-up analyses indicated that the number of back vowels was positively associated with memory for large definitions (*b* = 0.21, *t*[26] = 4.00) but negatively associated with memory for small definitions (*b* = –0.21, *t*[26] = –3.23). None of the other phonological features predicted memory performance (all *F* < 1.0, all *p* > 0.33). When examining memory for the size adjective only, none of the phonological properties predicted memory performance (all *F* < 2.9, all *p* > 0.10). The analyses on memory for the noun and adjective component mirrored those for the whole definition: the number of back vowels interacted with definition size (*F*(1, 23) = 7.62, *p* = 0.01, η^2^_p_ = 0.25). The number of back vowels was positively associated with memory for large definitions (*b* = 0.17, *t*[26] = 4.59) but negatively associated with memory for small definitions (*b* = –0.15, *t*[26] = –3.0).

In summary, both participant-level analyses, using experimenter-defined categories of large and small nonwords, and item-level analyses, using objective frequency of phonological properties, supported the hypothesis that congruency between sound symbolic properties and to-be-remembered information enhances memory performance.

### Experiment 2B – within-subjects design

The same analyses reported in Experiment 2A were performed; however, in this experiment, both factors were within-subjects in the participant analyses.

Looking at the total score, congruent items (*M* = 0.65, *SEM* = 0.42) were recalled more than incongruent items (*F*_*1*_(1, 73) = 35.71, η^2^_p_ = 0.33, *F*_*2*_(1, 26) = 31.34, η^2^_p_ = 0.55). Neither the effect of nonword size nor the interaction was reliable (*F*_*1*_(1, 73) = 1.08, *p* = 0.30, *F*_*2*_(1, 26) = 0.24, *p* = 0.62 and *F*_*1*_(1, 73) = 0.27, *p* = 0.60, *F*_*2*_(1, 26) = 0.78, *p* = 0.38, respectively). The analyses on recall for the size word revealed a main effect of congruency (*M*_*congruent*_ = 0.17, *M*_*incongruent*_ = 0.12) (*F*_*1*_(1, 73) = 9.45, η^2^_p_ = 0.12, *F*_*2*_(1, 26) = 6.59, *p* = 0.016, η^2^_p_ = 0.20), as well as a significant interaction (*F*_*1*_(1, 73) = 16.24, η^2^_p_ = 0.18, *F*_*2*_(1, 26) = 6.81, *p* = 0.015, η^2^_p_ = 0.21). The interaction reflected the fact that the congruency effect was not reliable for large nonwords (*M*_*congruent*_ = 0.13, *M*_*incongruent*_ = 0.13) (*t*(73) = 0.14, *p* = 0.89) but it was for small nonwords (*M*_*congruent*_ = 0.20, *M*_*incongruent*_ = 0.10) (t(73) = 4.38, Cohen’s *d* = 0.54). The effect of nonword size was not significant (*F*_*1*_(1, 73) = 2.51, *p* = 0.12, *F*_*2*_(1, 26) = 0.76, *p* = 0.39). Finally, the analyses on the adjective/noun component revealed an effect of congruency (*M*_*congruent*_ = 0.48, *M*_*incongruent*_ = 0.30) (*F*_*1*_(1, 73) = 36.83, η^2^_p_ = 0.33, *F*_*2*_(1, 26) = 32.83, η^2^_p_ = 0.56). Neither the effect of nonword size nor the interaction was reliable (*F*_*1*_(1, 73) = 0.08, *p* = 0.77 and *F*_*1*_(1, 73) = 2.22, *p* = 0.14, *F*_*2*_(1, 26) = 0.02, *p* = 0.89, respectively).

In the additional analyses at the item level, none of the phonological properties predicted memory performance (all *F* < 2.5, *p* > 0.13). When analyzing overall memory performance, the ANCOVA revealed a trend towards an interaction between the size implied by the definition and the number of back vowels (*F*(1, 23) = 2.47, *p* = 0.13, η^2^_p_ = 0.10). Follow-up analyses revealed the same pattern of results as those observed in Experiment 2A: a positive relationship between number of back vowels and memory for large definitions (*b* = 0.12, *t*[26] = 2.61, *p* = 0.01) and a negative relationship between number of back vowels and memory for small definitions (*b* = –0.12, *t*[26] = –2.34, *p* = 0.03).

### Discussion

Overall, congruent nonword and definition pairings were better remembered than incongruent pairings following a single study event. This occurred in both within- and between-subject designs. Furthermore, analyses focused on memory for size and memory for the adjective-noun components of the definition further supported the importance of congruency in both size symbolic characteristics (as evidenced by the mnemonic advantage for size information in congruent items) and in terms of the semantic appositeness of the nonword, which, as noted, was often related to the nonword in an onomatopoeic or associative fashion. This suggests that pairing nonword brand names with sound symbolically and semantically congruent products (where semantic refers to any meaning-based connection between the nonword and the definition) can enhance memory for these products. The only exception to the otherwise robust congruency effect was in the analysis on memory for size only in the between-subjects condition at the participant level where the effect was trending in the predicted direction. The item analyses indicated small effects of specific phonological properties on memory: the number of back vowels in a nonword was associated, albeit weakly, with memory. A congruency effect emerged between large definitions and nonwords with more back vowels, although such results might require cautious interpretation because of correlations between predictors.

## Experiment 3

Before concluding that congruency effects are robust in the present stimulus set, two potential and related issues need to be addressed. In Experiment 2, participants were told to remember the items for a memory test. Thus, it is likely they used some intentional or strategic encoding strategies, such as finding similarities or creating meaningful associations between the nonword and the definition. This use of top-down processes might have magnified the effects. To determine whether the congruency effect was driven by such intentional strategies, we administered the memory test using a modified stimulus set, described below, to two separate groups of participants. One group, the intentional learning group (Experiment 3A), was given identical instructions as participants in Experiment 2. The other group, the incidental learning group (Experiment 3B), was asked to rate each nonword-definition pair on how likely they would be to purchase a product with that name. This task was selected because it was assumed it would direct attention to both the nonword and the definition and require participants to make a decision based on both factors. However, no mention was made of a subsequent memory test.

In Experiment 2, the definitions paired with each nonword were derived from the descriptions provided by participants in Experiment 1B to those very nonwords. Thus, the robust congruency effect was potentially driven by similarity or relatedness between the nonword and the definition (e.g., *zrizz* was paired with *tiny child zipper* and *turob* with *large sports car*) that could have facilitated the associative learning process. For example, *zrizz* is reminiscent of the noise a zipper makes and *turob* is an orthographic neighbor to *turbo*. These items were congruent not only in terms of size, but potentially confounded in terms of associations between the nonword and the definition through mediators (e.g., *turbo* mediates between *car* and *turob*). Thus, the congruency effect might have been influenced by the fact that participants found it easier to create associations or use mnemonic devices based on the onomatopoeic properties of the definitions in the congruent condition. Consistent with this interpretation is the evidence that memory for congruent items was enhanced even when examining the adjective-noun components, which suggests that properties of the definition itself were in some way directly or indirectly associated with the nonwords. In the following studies, nonwords and definitions were re-paired to avoid this confound.

### Experiment 3A

#### Participants

Participants were recruited via Amazon’s Mechanical Turk. There were 84 participants, whose ages ranged from 21 to 68 years (*M* = 36.52, *SD* = 10.88) and whose mean education level ranged from 10 to 21 years (*M* = 15.22, *SD* = 2.20). Three participants (3.5%) were non-native speakers of English.

#### Materials

To examine the mnemonic effects of congruent size information only, we re-paired the nonwords and definitions such that the original definitions were now paired with other nonwords from the same size category to create congruent pairs (e.g., *zrizz* was paired with *tiny toilet plunger* and *turob* with *large flat disc*). Size-incongruent pairs were created by changing the size descriptor (e.g., *zrizz* was paired with *large toilet plunger* and *turob* with *small flat disc*). Thus, congruency was now solely in terms of the size conveyed by the nonword and the size of the object described.

The same 28 nonwords used in Experiment 2 were used (14 large and 14 small). Across items, congruency was counterbalanced across participants.

#### Procedure

The procedure was identical to that used in Experiment 2.

#### Results

**Fig. 2:**
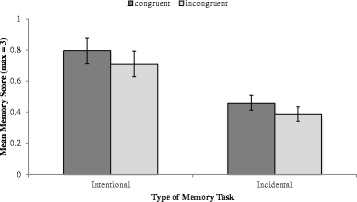
Mean memory score out of three possible points as a function of congruency in Experiments 3A (intentional encoding) and 3B (incidental encoding). Error bars reflect the standard error of the mean

Memory performance was scored as in the previous experiment (see Fig. 2). The analyses using experimenter-defined size categories indicated a non-significant effect of congruency (*F*_*1*_(1, 83) = 2.76, *p* = 0.10, η^2^_p_ = 0.03, *F*_*2*_(1, 26) = 1.23, *p* = 0.28). Albeit not significant, the direction of the effect was consistent with the other analysis, such that congruent items were recalled slightly more accurately than incongruent items (*M*_*congruent*_ = 0.68, *M*_*incongruent*_ = 0.64). The effect of nonword size was marginally significant (at the participant level) (*F*_*1*_(1, 83) = 3.32, *p* = 0.07, η^2^_p_ = 0.04, *F*_*2*_(1, 26) = 1.63, *p* = 0.21), such that definitions paired with small nonwords were recalled better than those paired with large nonwords (*M* = 0.69 and *M* = 0.64, respectively). The interaction was not reliable (*F*_*1*_(1, 83) = 2.76, *p* = 0.10, *F*_*2*_(1, 26) = 0.02, *p* = 0.90). When memory for the size descriptor alone was analyzed, the congruency effect persisted in the analyses by participants (*F*_*1*_(1, 83) = 5.96, *p* = 0.02, η^2^_p_ = 0.07, *F*_*2*_(1, 26) = 1.98, *p* = 0.17) (*M*_*congruent*_ = 0.23, *M*_*incongruent*_ = 0.19). The effect of nonword size and the interaction were not significant (*F*_*1*_(1, 83) = 0.20, *p* = 0.65, *F*_*2*_(1, 26) = 0.05, *p* = 0.82 and *F*_*1*_(1, 83) = 2.76, *p* = 0.10, *F*_*2*_(1, 26) = 1.28, *p* = 0.27, respectively). Finally, the analyses on the other components of the definitions revealed that, consistent with the previous analyses, definitions associated with small nonwords (*M* = 0.47) were remembered better than those associated with large nonwords (*M* = 0.42) (*F*_*1*_(1, 83) = 5.14, *p* = 0.03, η^2^_p_ = 0.06, *F*_*2*_(1, 26) = 2.26, *p* = 0.14). Neither the effect of congruency nor the interaction were significant (*F*_*1*_(1, 83) = 0.21, *p* = 0.65, *F*_*2*_(1, 26) = 0.12, *p* = 0.74 and *F*_*1*_(1, 83) = 0.90, *p* = 0.34, *F*_*2*_(1, 26) = 0.69, *p* = 0.42, respectively).

Item analyses with phonological properties as predictors did not yield any significant effects in terms of overall memory performance (all *F* < 2.6, all *p* > 0.12) or in terms of memory for the size component (all *F* > 1.10, all *p* > 0.30). However, the analyses on memory for the adjective and noun components of the definition revealed two interactions with phonological properties: the number of plosives (*F*[1, 23] = 4.37, *p* = 0.048, η^2^_p_ = 0.16) and the number of small vowels (*F*[1, 23] = 4.96, *p* = 0.036, η^2^_p_ = 0.18) interacted with the implied size of the definition. Follow-up analyses on the interactions indicated that the number of plosives was negatively related with memory for small definitions (*b* = –0.08, *t*(26) = –2.68, *p* = 0.013), whereas the number of plosives and memory for large definitions were unrelated, (*b* = –0.03, *t*(26) = –0.97, *p* = 0.34). The follow-up analyses on the interaction between number of front vowels and definition size indicated no systematic relationship (for large definitions, *b* = 0.04, *t*[26] = 1.56, *p* = 0.13; and for small definitions, *b* = 0.01, *t*[26] = 0.34, *p* = 0.74). Given that the adjective and noun component of the definitions were intentionally arranged to minimize any influence of semantic appositeness or onomatopoeia, such results are likely to be due to random or uncontrolled factors.

Thus, when potential contributions of associations driven by factors such as onomatopoeic content or mediated associations between the nonword and the definition are removed, the congruency between sound symbolic properties of nonwords and object descriptions seems to be limited to enhanced memory for the size of the object only, and not for other characteristics of the object. None of the individual phonological properties accounted for this effect; although experimenter-defined classification of nonwords in terms of size did yield a reliable congruency effect in memory performance, it is not clear, from the present stimulus set, which unique factors or properties of the nonwords are driving the effect.

### Experiment 3B

#### Participants

One hundred and twelve native English speaking participants from Mechanical Turk were recruited. Participants’ age ranged from 20 to 68 years (*M* = 35.67, *SD* = 10.80) and their reported years of education ranged from 12 to 25 (*M* = 16.67, *SD* = 2.54).

#### Materials

The materials were the same as Experiment 3A.

#### Procedure

The procedure was similar to Experiment 3A, but instead of the nonword and its three-word definition appearing on the screen for 8 s, participants used a slider scale to rate how likely they would be to buy the “product” as it was defined and then clicked an icon on the computer screen to progress to the next nonword-definition pairing. The scale ranged from 0–10, zero meaning the participant definitely would not buy the product and 10 meaning the participant certainly would buy the product. Participants were not told about the memory test until after completing the study phase.

#### Results

Under incidental learning conditions, overall performance was substantially lower. In the analyses examining congruency based on experimenter-defined categories, a reliable effect of congruency (*F*_*1*_(1, 111) = 5.57, *p* = 0.02, η^2^_p_ = 0.05, *F*_*2*_(1, 26) = 3.67, *p* = 0.07, η^2^_p_ = 0.12) (*M*_*congruent*_ = 0.35, *M*_*incongruent*_ = 0.30) was present. The effect of nonword size was also reliable (*F*_*1*_(1, 111) = 30.75, η^2^_p_ = 0.22, *F*_*2*_(1, 26) = 6.20, *p* = 0.02, η^2^_p_ = 0.19) (*M*_*small*_ = 0.38, *M*_*large*_ = 0.27). The interaction was not significant (*F*_*1*_(1, 111) = 1.17, *p* = 0.28, *F*_*2*_(1, 26) = 0.79, *p* = 0.38). In the analyses on memory for the size descriptor, there was a main effect of congruency (*M*_*congruent*_ = 0.14, *M*_*incongruent*_ = 0.09) (*F*_*1*_(1, 111) = 13.70, η^2^_p_ = 0.11, *F*_*2*_(1, 26) = 8.97, η^2^_p_ = 0.26), a main effect of nonword size (*M*_*small*_ = 0.38, *M*_*large*_ = 0.27) (*F*_*1*_(1, 111) = 8.55, η^2^_p_ = 0.07, *F*_*2*_(1, 26) = 1.76, *p* = 0.20, η^2^_p_ = 0.06), and an interaction (*F*_*1*_(1, 111) = 5.48, *p* = 0.02, η^2^_p_ = 0.05, *F*_*2*_(1, 26) = 3.48, *p* = 0.07, η^2^_p_ = 0.12). The interaction was given by the fact that size descriptors were recalled more accurately for congruent small than congruent large items (*M* = 0.17 and 0.11, respectively) whereas there was no difference for incongruent items (both *M* = 0.09). Turning to the adjective and noun components, analyses revealed that the congruency effect was driven by the size descriptor, as it was absent in the rest of the definition (*F*_*1*_(1, 111) = 0.07, *p* = 0.79, *F*_*2*_(1, 26) = 0.04, *p* = 0.84) (*M*_*congruent*_ = 0.21, *M*_*incongruent*_ = 0.21). Definitions paired with small nonwords were remembered better than those paired with large nonwords (*F*_*1*_(1, 111) = 25.29, η^2^_p_ = 0.19, *F*_*2*_(1, 26) = 5.92, *p* = 0.02, η^2^_p_ = 0.18). The interaction was not significant (*F*_*1*_(1,111) = 0.13, *p* = 0.72, *F*_*2*_(1, 26) = 0.07, *p* = 0.79).

The analyses with the phonological predictors, as in Experiment 3A, did not yield any reliable effects for overall memory (all *F* < 2.6, *p* > 0.12) or memory for the size term (all *F* < 2.81, *p* > 0.10). Consistent with Experiment 3A, marginal interactions between the size implied by the definition and the number of plosives emerged (*F*(1, 23) = 3.45, *p* = 0.08, η^2^_p_ = 0.13), and between the size implied and the number of front vowels (*F*(1, 23) = 3.24, *p* = 0.08, η^2^_p_ = 0.12). The number of plosives was negatively associated with memory adjective and noun components for small definitions (*b* = –0.08, *t*(26) = –2.41, *p* = 0.02), whereas the relationship between number of plosives and memory for adjectives and nouns from large definitions was non-significant (*p* = 0.22). Follow-up analyses on the relationship between front vowels and memory performance were non-significant (both *p* > 0.20).

Thus, consistent with the results of Experiment 3A, once the relationship between the nonword and the paired definition is congruent only in terms of size, any effect of sound symbolism on memory is reduced to memory for the size information alone. Importantly, this enhanced memory occurred even in the absence of intentional learning processes, suggesting that the sound symbolic information that facilitates learning and retention does not exclusively depend on the engagement of top-down, strategic encoding processes. Under incidental encoding conditions, the key effects were reduced, suggesting that some implicit learning or associations might underlie how sound symbolism can influence learning.

## Experiment 4

In the final study, we examined whether the congruency effects observed in previous studies were driven by the indirect activation of the word neighbors of the nonwords. Because all nonwords had at least one word neighbor (as defined by Coltheart et al.’s, 1977 metric of a single substitution), one possibility is that the congruency effects in Experiments 2 and 3 and the size effect in Experiment 1 were due to participants making assessments of size based on a word neighbor. In other words, when asked to make a size judgment of an item with no known referent, it is plausible that participants relied on their knowledge of an object with a similar name or sound to the nonword. For example, the nonword *blomp* might have been classified as large because of its neighbor *blimp*. The fact that the congruency effect for components of the definition other than size was absent in Experiment 3 when definitions and nonwords were recombined does suggest that the ease of associating a definition with a nonword seems to depend on the content of the definition itself. Recombined definitions likely increased the difficulty of finding an association or mnemonic cue between the nonword and the definition; thus, the mnemonic advantage disappeared.

Furthermore, nonwords do prime their word neighbors and associates (e.g., Kinoshita & Norris, 2009; Perea & Lupker, 2003) suggesting that a similar process might be at work here (i.e., an automatic activation process that increased the accessibility of *blimp* in semantic memory once *blomp* was presented and that biased participants to assess *blomp* as large). To confirm that it was the sounds of the nonwords themselves which elicited associations of size and not their real word neighbors, participants completed a free association task in which they were asked to generate the first word that came to mind in response to the nonwords, and responses were coded for size. If the size of the word neighbor influenced how participants rated and processed the nonwords, one would expect that large nonwords would give rise to more responses classified as large than small, and vice versa for small nonwords. Thus, an interaction between nonword size and the size of the object given by participants was the critical result for which we tested. As in Experiments 1A and 1B, we used a standard shoe box as a referent, classifying objects that could fit in a shoe box as small (e.g., *glasses*, *medicine*), and large as those objects that, in their typical form, would not (e.g., *blanket*, *car*). In cases of multiple senses, such as a response being a verb or a noun, we used the noun meaning. All responses that referred to a person, such as *judge*, were classified as large. Although the free associate responses given by participants might have been orthographically or phonologically related, our main interest was in whether they were large or small objects. Thus, we did not code whether the responses were related or unrelated to the cues, but only whether the responses referred to large or small referents.

### Experiment 4A

The first version of the free association task was unconstrained; participants were simply asked to write the first word that came to mind in response to the nonword.

#### Participants

Forty-five participants were recruited from Mechanical Turk. Due to a programming error, data from five participants were lost; thus, the analyses include data from 40 participants, two of whom (5%) were non-native speakers of English.

#### Materials and procedure

The 28 nonwords used in Experiments 2 and 3 were used. Stimulus presentation was implemented using Qualtrics online survey software. Each nonword was presented individually with a text box and participants were asked to type a single word in response to the nonword. Instructions stated they should report the first word that came to mind and that there were no right or wrong answers. All 28 nonwords were presented in a new random order for each participant.

#### Results

In both experiments 4A and 4B, the first author coded the responses. Overall, the majority of responses (64.4%) could not be classified in terms of size, because they referred to abstract concepts (e.g., *ability*, *rarity*), were adjectives (e.g., *glad*, *thankful*), verbs (e.g., *sneeze*, *swat*), or other parts of speech (e.g., *again*, *next*). Item analyses were conducted on the proportion of large and small responses to each nonword, with nonword size as a between-items factor and response as a within-items factor. Only the effect of nonword size approached significance (*F*_*2*_(1, 26) = 3.97, *p* = 0.06, η^2^_p_ = 0.13), with large nonwords eliciting more “sized” responses than small nonwords (*M* = 0.22, *SEM* = 0.03 and *M* = 0.13, *SEM* = 0.03, respectively). Neither the effect of given response size nor the interaction were significant (*F*_*2*_ (1, 26) = 0.75, *p* = 0.39 and *F*_*2*_ (1, 26) = 0.26, *p* = 0.61, respectively). Thus, the size judgments provided in the previous studies did not seem to be mediated by word neighbor information. However, because the present analyses depend on accepting a null hypothesis, we additionally performed a Bayesian analysis. We used JASP software (JASP version 0.8 beta 5) and the default settings (Wagenmakers et al., under review). The default settings of JASP assign Cauchy priors to effect sizes (all models are given equal probability). The results indicated that the data were more likely to occur under the null hypothesis, with a Bayes Factor (BF_01_; null/hypothesis) for the critical interaction of 6.27 (i.e., the data were more likely to occur under the null hypothesis).

### Experiment 4B

Given the large number of responses that were impossible to code for size (i.e., verbs, adjectives, adverbs), the instructions were slightly modified to encourage participants to think of a noun rather than a descriptor such as an adjective.

#### Participants

Seventy-three participants from the same pool were recruited. Because of the large number of invalid responses in Experiment 4A, we increased the sample size. Two participants (2.7%) were non-native speakers of English.

#### Materials and procedure

Other than the modification to the instructions to try to elicit more noun responses, the experiment was identical to Experiment 4A.

#### Results

The instructional change was moderately successful. Overall, 50% of responses were coded as large or small. The proportions of size responses were submitted to the same analysis as in Experiment 4A. Once again, large nonwords were more likely to elicit a sized response (*M* = 0.29, *SEM* = 0.02) than small nonwords (*M* = 0.21, *SEM* = 0.02) (*F*_*2*_(1, 26) = 6.74, *p* = 0.015, η^2^_p_ = 0.21). However, neither the effect of given size nor the interaction were reliable (*F*_*2*_ (1, 26) < 0.001, *p* ≥ 0.99 and *F*_*2*_ (1, 26) = 1.58, *p* = 0.22, respectively). As in Experiment 4A, the same Bayesian analysis confirmed that the observed results were more likely to occur under the null, as indicated by a BF_01_ (null/hypothesis) of 3.68.

#### Discussion

The results of both Experiments 4A and 4B suggest that it is unlikely that large and small nonwords elicited sound symbolic size effects due to mediation from large or small word neighbors. In other words, the large and small nonwords did not seem to be strongly associated with large or small objects in a way that would drive the effects observed.

## General discussion

Using a large set of novel nonwords selected using known phonological distinctions to elicit perceptions of largeness or smallness, we found evidence for sound symbolism effects in a number of tasks. First, sound symbolism effects were found using a continuous scale of size judgment rather than a binary categorization task (i.e., small or large?). These results support and extend upon the results of Klink (2000) who found sound symbolism effects for nonwords containing stops and fricatives in a forced choice task where participants only had a limited number of answer choices to associate with a given nonword. Notably, the nonwords used in the present study reflected natural language in their variability, such that although we relied on pre-existing distinctions in the literature, there was intra-categorical similarity between small and large nonwords. Even under these conditions, with a less extreme class of stimuli, reliable effects of size sound symbolism emerged.

In Experiment 1B, some spontaneously generated sound symbolism effects were evident: small nonwords elicited definitions referring to small objects more than large nonwords, and the opposite was also observed. Furthermore, definitions of large nonwords were more likely to include the word *large* or related words than small nonwords. These findings are potentially relevant for marketing research that seeks to create brand names that can be readily associated with a target product. Analyzing the data differently, by ranking a subset of each participant’s responses by size, yielded similar effects suggesting the magnitude effect is not dependent on the coding scheme applied to the data. Although these results are promising, the instructions given in this study may have limited participant responses by biasing them towards smaller products. Therefore, further research using different instructions and new nonwords is needed. Furthermore, in the present study we focused on size. As Klink (2000) reported, different sound categories are also associated with characteristics such as femininity, softness, lightness, and so on. Thus, it is possible that a new product name can be developed in a manner that facilitates the formation of connections between the name and the product through sound symbolic effects.

In Experiment 2, a congruency effect was found in the memory task for the participant-generated semantically apposite three-word definitions of the nonwords; congruent nonword-definition pairs were recalled more often than incongruent pairs. This effect was found not only for the whole three-word definition, but also the separate size and adjective-noun components of the definition. The congruency effect, however, did not fully emerge in Experiment 3 when the nonwords and their definitions were recombined. Under those conditions, only the size component of the definitions seemed to drive the congruency effect. This suggests that, in Experiment 2, in addition to congruency between the sound qualities of the nonwords and the selected size descriptor, some associative benefit or mnemonic advantage between the adjective-noun component of the definition and the nonword was also present. Whereas the former is more likely to have been driven by sound symbolic properties, we suggest the latter was due to higher-order or top-down processes. Specifically, onomatopoeic or object-sound associations that were present in some of the nonword-definition pairings (e.g., *zrizz*-*zipper*) likely afforded the application of strategic mnemonic strategies. Taking away these potential associative or semantic contributions in Experiment 3 revealed that size congruency effects persisted without it, but were quite small and, under incidental learning conditions in Experiment 3B, were restricted to the size descriptor. Taken together, the results of Experiments 2 and 3 suggest that different processes drive the congruency effects for size symbolic properties and semantic or conceptual content. Sound symbolism effects in memory may be boosted by semantics, but are not exclusively driven by them. Most importantly, the sound symbolic properties appear to exert a mnemonic advantage that does not depend on intentional learning strategies or elaborative encoding.

The results from the memory studies are consistent with work by Parault and Parkinson (2008, 2006); Parault and Schwanenflugel (2006) who reported that the meanings of novel words were learned more effectively when there were sound symbolic relations between the words and the to-be-learned meaning. In Parault et al.’s studies, participants initially guessed the meaning of obscure words and then completed a recognition task. Although participants in their study were not given explicit memory instructions, the fact that it was a word learning task and that they were instructed to try to learn new word meanings might have elicited some more strategic processing. Here, we extended these results to intentional and incidental memory for nonword-definition pairs, which is a conceptually similar task to vocabulary learning. Prior associative learning studies have used shape categories (e.g., curvy vs. angular). Aveyard (2012) observed that associations between novel shapes and nonwords were facilitated depending on the sounds in the nonwords. Curvilinear shapes were learned better when paired with nonplosive nonwords (e.g., *fuh-li-sai*), whereas rectilinear shapes were learned better with plosive nonwords (e.g., *kuh-der-pai*). However, other studies (Monaghan et al. 2012) suggest this boost in associative learning is restricted to category learning, such as curvy vs. angular shapes, rather than the learning of individual words or objects. The fact that congruent sound symbolic size information was remembered better than other aspects of the definition when the definitions were recombined might be interpreted as evidence that size information serves as a way of categorizing the items.

One possible explanation for the observed results is that meaning-based processing of nonwords was the driving force of sound symbolism. Physical size information can be activated when processing words. Rubinsten and Henik (2002) found that the real-life size of animals interfered with participants’ judgments of font size of the written animals’ names (i.e., the font size of the word). Similarly, Setti et al. (2009) found that target words were easier to categorize if a same real-life size prime preceded the target word. Sereno et al. (2009) found that participants were faster in a lexical decision task when words represented large objects (e.g., *bookcase*) verses small objects (e.g., *teaspoon*). Thus, size may be automatically activated when processing words and can yield congruency effects.

Experiment 4 allowed us to test one direct role for meaning-based processing of nonwords. If word neighbors of the nonwords were systematically confounded with the nonword size classification, these neighbors may have been driving sound symbolism effects. However, this did not seem to be the case. The free associations generated in response to the nonwords were not words that described small or large objects. This suggests that direct or indirect association with sized word referents does not uniquely drive sound symbolism. Together, these studies suggest that processing of word meaning elicits sensorimotor experiences with the word. We suggest that the sounds included in the nonwords can also elicit similar sensorimotor experiences specific to size and that these associations can promote the formation of associations in memory.

The previous conclusions are based primarily on the results of the analyses using congruency between experimenter-defined nonword size classifications and the size of the referent in the definitions. Although the analyses examining the role of specific phonological predictors of sound symbolic effects did not yield consistent or robust findings, we argue our conclusions are warranted for two reasons. First, the rating data of Experiment 1A did confirm our selection of stimuli in that our small and large nonwords were, indeed, rated as small and large. Second, the analyses based on phonological properties, as noted, might have been limited by the relatively high inter-correlations between properties. Because the stimuli contained multiple phonological properties associated with size, it is possible that the memory effects, which are relatively small, are not driven by one specific property. Rather, additive effects of different classes of phonological features might be necessary to influence memory performance, at least when additional semantic or onomatopoeic information is not available. This suggests that, for example, a nonword that only contains one feature associated with size (e.g., a front vowel) might not yield sound symbolic effects unless it also contains other phonemes associated with size (e.g., one or more plosives). Clearly, there is a need for further research to focus more specifically on which phonological features drive sound symbolic effects.

These findings, especially those of Experiments 2 and 3, have important implications in the advertising and the marketing of products. When creating nonword brand names of new products, companies should consider the effects that sound symbolism may have on a consumer’s associations and memory for the product. If brand names include phonemes congruent with the size of the product according to the rules of sound symbolism, consumers may better remember the product and its name. Experiments 2 and 3 also suggest that including some information that facilitates the formation of a meaning-based association in the nonword brand name can aid in memory for a product (e.g., the brand name *Zrizz* for a zipper). Such findings are consistent with earlier work on the effects of semantic appositeness (Lowrey et al., 2003); they add to this literature evidence that even semantic or perceptual information, such as an object’s size, can be activated by the phonetic properties of elements comprising nonwords.

The above suggestions for the creation of brand names are in line with existing recommendations for brand name development. Certain nonwords can bias a certain meaning or association with different product categories, making them more appropriate for some products than others. For example, among computer-generated nonwords, the nonword *whumies* was more appropriate as a cereal brand name than a detergent brand name (Peterson & Ross, 1972). Even nonsensical alpha-numeric strings have shown to be more appropriate for technical products than non-technical products (Pavia & Costa, 1993), once again demonstrating people’s search for meaning in nonsensical or random letter/number strings. Because meaningful associations, those that are driven by some reliance on sound characteristics even in the context of nonwords, were easier to remember than non-meaningful associations, it would be beneficial to include some intended semantic meaning in brand names rather than make a brand name without considering these effects. The results of the present study further suggest that size information, implied by specific sound categories, contributes additional memorability to nonword-definition pairings.

## Conclusions

In a total of four experiments, several conclusions were drawn regarding the strength of sound symbolism effects and the role they play in associative memory. Experiment 1 replicated the results of previous research in that nonwords composed of “small” phonemes (small nonwords) were rated as smaller than those composed of “large” phonemes (large nonwords), but with a more variable set of stimuli that better simulated naturally occurring language and on a continuous size scale for participant size judgment. This supports the existence of sound symbolism effects outside of an experimental setting or forced choice task. The results of this experiment prompted us to proceed with the following ones. In Experiments 2 and 3, it was concluded that sound symbolism could boost memory for nonword-definition parings, with or without any mnemonic advantage the participant-generated definitions may have provided in Experiments 2A and 2B. These results have important implications for marketing teams seeking to create memorable nonword brand names as they suggest that sound symbolism plays a role in associative memory. Finally, in Experiment 4, a final free association study was conducted to rule out the possibility that large and small nonwords elicit sound symbolic size effects due to mediation from word neighbors. This ensured that our previous results were driven by sound symbolism and, once again, suggest that this phenomenon is a factor when processing nonwords.

## Appendix 1

**Table 4:** List of 100 novel nonword stimuli utilized in Experiment 1 and their average size scores from Experiment 1A

Small nonwords		Large nonwords	
Nonword	Average size score	Nonword	Average size score
adiea	42.11	afoub	44
**afain**	35.43	**agort**	53.9
afent	35.44	agout	50.35
afree	39	anour	43.93
afrid	39.15	antuc	46.36
afrox	38.84	atolp	42.33
ahaze	47.13	**bloff**	58.58
alape	47.51	**blomp**	68.89
**ality**	33.75	bomeo	56.94
**almit**	33.81	**borno**	58
alvil	40.14	chrub	38.43
**anibi**	26.11	chrug	45.88
anlas	40.15	dahoo	43.9
**arity**	36.4	dlurb	44.89
assiy	33.7	dolch	45.09
assoy	42.13	ducab	44.44
atble	40.2	duldo	47.27
avice	42.24	dumob	58.16
avise	46.44	dutal	43.46
avity	41.59	furob	43.63
bicel	43.13	gloch	58
blief	42.21	**glurb**	53.93
cilia	24.69	glurp	48.78
clafa	47	**glutu**	62.82
**clish**	27.1	**gnomp**	52.44
**eevie**	20.38	**golon**	65.06
**feese**	37.26	**gruse**	56.1
fiata	54.56	gurro	42.32
frasl	41.15	hebop	45.66
frool	40	hobop	49.69
fyral	43.35	honob	50.2
helra	50.69	**jolge**	59
**iviel**	31.67	klurb	47.89
lrizz	37.42	koubt	45.16
lyffs	39.9	mooep	46.16
**nelch**	28.64	nogom	46.21
nevil	38.6	nosom	41.89
nixin	36.82	**phomp**	60.76
rirca	42.81	pluko	42.5
**rivic**	35.79	scrog	49.05
salva	45.48	thoap	42.23
**sless**	29.26	thoot	42.19
srizz	37.4	**thuap**	51.97
vilve	41.1	**thuop**	66.7
vimms	44.16	tlurb	46.12
virca	40.38	tocoa	45.6
**viscs**	32.39	tooby	49.91
vitav	40.56	troog	51.7
vrizz	39.3	**turob**	54.63
**zrizz**	28.41	uppun	50.16

*Note.* Bold nonwords compose the 28 nonwords included in Experiment 2

## Appendix 2

**Table 5:** Nonwords and definitions used in Experiments 2 and 3

Nonword size category	Nonword	Size-congruent definition	Size-incongruent definition
Experiment 2			
large	agort	large gel tube	small air spray
large	bloff	large puffy clothing	small winter clothing
large	blomp	large soft chair	Small electrical gadget
large	borno	bulky laundry detergent	portable smooth lotion
large	glurb	giant green drink	little shiny makeup
large	glutu	huge exercise equipment	small kitchen cleaner
large	gnomp	large gardening tool	warm small gloves
large	golon	large dark liquid	little spiky toy
large	gruse	big grease cleaner	small rugged razor
large	jolge	giant dishwasher detergent	small metal tool
large	phomp	large toilet plunger	tiny child zipper
large	thuap	big fly swatter	tiny pain medication
large	thuop	big cleaning mop	small light shoes
large	turob	large sports car	small flat disc
small	afain	tiny pain medication	big fly swatter
small	ality	little shiny makeup	giant green drink
small	almit	warm small gloves	large gardening tool
small	anibi	little spiky toy	large dark liquid
small	arity	small air spray	large gel tube
small	clish	small kitchen cleaner	huge exercise equipment
small	eevie	small electrical gadget	large soft chair
small	feese	small light shoes	big cleaning mop
small	iviel	portable smooth lotion	bulky laundry detergent
small	nelch	small metal tool	giant dishwasher detergent
small	rivic	small rugged razor	big grease cleaner
small	sless	small winter clothing	large puffy clothing
small	viscs	small flat disc	large sports car
small	zrizz	tiny child zipper	large toilet plunger
Experiment 3			
large	agort	large air spray	small air spray
large	bloff	large winter clothing	small winter clothing
large	blomp	large electric gadget	small electrical gadget
large	borno	bulky smooth lotion	portable smooth lotion
large	glurb	giant shiny makeup	little shiny makeup
large	glutu	huge kitchen cleaner	small kitchen cleaner
large	gnomp	warm large gloves	warm small gloves
large	golon	large spiky toy	little spiky toy
large	gruse	big rugged razor	small rugged razor
large	jolge	giant metal tool	small metal tool
large	phomp	large child zipper	tiny child zipper
large	thuap	big pain medication	tiny pain medication
large	thuop	big light shoes	small light shoes
large	turob	large flat disc	small flat disc
small	afain	tiny fly swatter	big fly swatter
small	ality	little green drink	giant green drink
small	almit	small gardening tool	large gardening tool
small	anibi	little dark liquid	large dark liquid
small	arity	small gel tube	large gel tube
small	clish	small exercise equipment	huge exercise equipment
small	eevie	small soft chair	large soft chair
small	feese	small cleaning mop	big cleaning mop
small	iviel	portable laundry detergent	bulky laundry detergent
small	nelch	small dishwasher detergent	giant dishwasher detergent
small	rivic	small grease cleaner	big grease cleaner
small	sless	small puffy clothing	large puffy clothing
small	viscs	small sports car	large sports car
small	zrizz	tiny toilet plunger	large toilet plunger

## Footnotes

1 The different standard for appliances was used when participants described something as a “small kitchen appliance” because, although many kitchen appliances are larger than a shoebox, a number of them are smaller than the “average” or typical appliance, which is, based on free association frequency in the University of South Florida Free Association Norms (Nelson, McEvoy, & Schreiber, 1998), a dishwasher.

2 In the following experiments, sample size varied based on participant availability on Mechanical Turk after a minimum of 60 participants were tested.

3 We thank an anonymous reviewer for this suggestion.
